# Temporal Transcriptional Responses of a Vibrio alginolyticus Strain to *Podoviridae* Phage HH109 Revealed by RNA-Seq

**DOI:** 10.1128/msystems.00106-22

**Published:** 2022-04-11

**Authors:** Xixi Li, Ce Zhang, Xingkun Jin, Fucheng Wei, Fei Yu, Douglas R. Call, Zhe Zhao

**Affiliations:** a Department of Marine Biology, College of Oceanography, Hohai University, Nanjing, China; b College of Oceanography, Hohai University, Nanjing, China; Tufts University

**Keywords:** bacterial lysis, lytic phage, RNA-seq, transcriptomic regulation, *Vibrio alginolyticus*

## Abstract

Phage are thought to exhibit control over host genes during infection. As a preliminary investigation of the kinetics and magnitude of co-expression between phage and bacteria, we compared the global transcriptional profiles for Vibrio alginolyticus strain E110 and its lytic phage HH109 by using RNA sequencing. In total, 24.7% (1,143/4,620) of the host protein-coding genes were differentially expressed genes during infection (DEGs). Functional analysis of the host DEGs suggests that phage HH109 induced rapid and distinctive changes when compared with 60- and 120-min postinfection (mpi). Based on gene co-expression network analysis, an uncharacterized late gene *gp27* encoded by the phage HH109 was predicted to modulate the host’s membrane transport and/or transcriptional regulation. Furthermore, expression of several bacterial virulence genes was downregulated while drug resistance genes were upregulated. This work contributes to an in-depth understanding of the reciprocal interactions of lytic phage HH109 and its pathogenic *Vibrio* host E110, and can provide new insights into the research and development of phage therapy against pathogenic *Vibrio* infections in the economically significant aquatic animals.

**IMPORTANCE** Vibrio alginolyticus is a common opportunistic pathogen that causes mass mortality in cultured marine animals. Phage HH109 lyses pathogenic V. alginolyticus strain E110 with high efficiency and thus serves as a useful model to understand the dynamic interplay of a phage and its host. Global transcriptomic responses of strain E110 post-HH109 infection were characterized by using RNA sequencing, elucidating step-by-step control by HH109, an antiphage-like responses, and the elevated expression of drug resistance. This study provides a detailed molecular description phage and V. alginolyticus, providing insight into better prevention and control of vibriosis in aquatic animals.

## INTRODUCTION

Vibrio alginolyticus, a Gram-negative opportunistic pathogen of marine aquatic organisms, is one of the causative agents of epidemic vibriosis that causes mass mortality of cultured fish, shellfish, shrimp, and coral reefs (1). And while uncommon, V. alginolyticus can cause otitis, wound, and intestinal infections in people (2). Treatment usually involves macrolide, tetracycline, or beta-lactam antibiotics for both medical and veterinary cases (3). Multidrug-resistant strains of V. alginolyticus have recently emerged across different marine ecosystems, including an offshore site, aquaculture environment, and water sediment (4, 5). We isolated V. alginolyticus strain E110 from the hepatopancreas of diseased white leg shrimp (Penaeus vannamei). E110 harbors virulence genes including a metalloprotease (*mtp*), alkaline serine protease (*asp*), type III secretion system (T3SS), and thermostable direct hemolysin (*tdh*).

Phage are viruses that can specifically recognize and infect a bacterial host and are found ubiquitously in many environments (6). More than a century ago, phage was used to treat bacterial infections (7), but the invention, ease of use, and ultimate adoption of antibiotics overshadowed the therapeutic potential of bacterial phage in the most parts of the world (8). With the growing crisis of antibiotic resistance, there is a renewed interest in using phage as therapeutics (9). Phage can be very selective with respect to susceptible strains, which is unique from the less specific activity of traditional antibiotics (10). Nevertheless, phage therapy can be limited by relatively narrow host range even within species, and the ligand-specific recognition mechanism is susceptible to resistance from spontaneous mutations (11).

Like all viruses, phage rely on host cell molecular and biochemical systems for reproduction prior to lysis (12). Lytic phage undergo adsorption to a cell surface, injection across the cell membrane, genome replication, transcriptional translation, virion assembly, and release to complete the infection cycle (13). Phage have developed various strategies to facilitate the release of virion progeny from infected host bacterial cells. Among them, the holin-endolysin system serves as a model for most dsDNA lytic phage (14), where holin-like proteins induce the formation of holes in the cytoplasmic membrane at a precise time to release the endolysin that accumulates in the host cell cytoplasm, resulting in cell burst (15). In addition, phage can use bacterial secretion systems to release endolysin that causes cell membrane depolarization, which in turn facilitates the endolysin-mediated cell lysis (16). Bacteria have also evolved a wide range of defense mechanisms against phage infection, including restriction-modification (RM) systems, “clustered regularly interspersed palindromic repeats” (CRISPR) loci together with CRISPR-associated (Cas) genes, and the abortive infection (Abi) system (17, 18). The process of phage-host interaction is a complex and dynamic arms race that is diverse in co-evolved interactions (19). Escherichia coli phage (20) and Pseudomonas aeruginosa phage (21) have served as our primary models for understanding host-phage interactions, but this limited scope may obscure understanding that could lead to improved phage therapy.

Previously, we isolated and identified a novel phage that was designated as HH109. This phage is a member of the *Podoviridae* family and it exhibits efficient lytic activity against V. alginolyticus strain E110. HH109 shares 99% sequence similarity with another phage VP670 (GenBank: KY290756), that was originally isolated from a different strain of V. alginolyticus (22). They both harbor 49 open reading frames (ORFs) but functional analysis has been limited to the holin and endolysin proteins (23). In this study, time-series RNA sequencing (RNA-seq) was employed to investigate the global effect of phage HH109 on the V. alginolyticus transcriptome. A myriad of differentially expressed genes (DEGs) were identified after phage infection, and gene-gene interaction networks provide insight into how the host and pathogen have co-evolved over time.

## RESULTS

### Lysis profiles of phage HH109 against V. alginolyticus.

**FIG 1:**
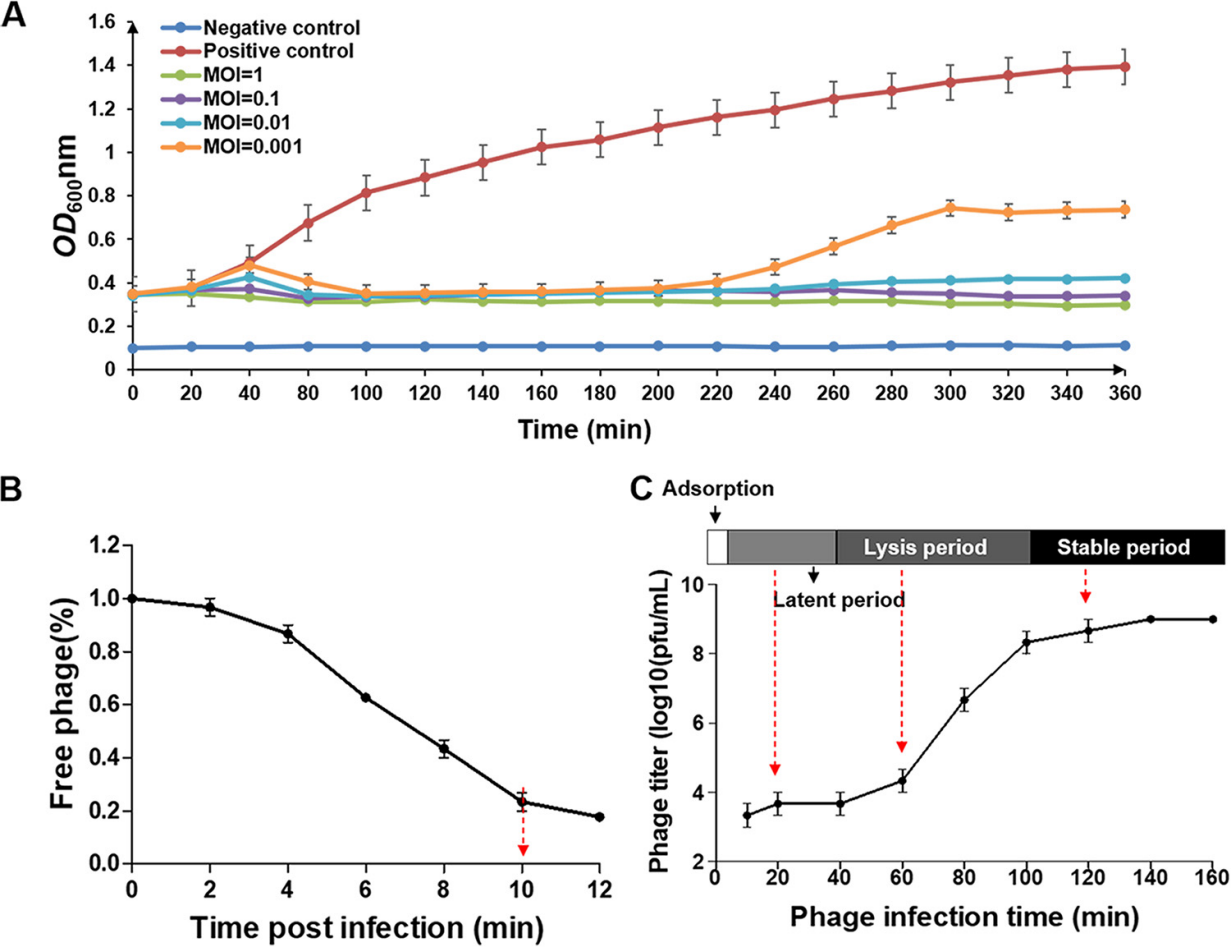
Infection dynamics of V. alginolyticus E110 by phage HH109. (A) Phage-mediated lysis of bacterial cultures at various MOI. Lysis was monitored by measuring the OD_600_values for samples taken at the indicated times after infection. (B) Adsorption curve of HH109 to its host E110. The *x* axis shows the incubation time of HH109 and its host, and the *y* axis shows the percentage of the phage that did not adsorb to the host. (C) One-step growth curve of phage HH109. The *x* axis shows the incubation time of HH109 with its hosts after absorption for 10 min. The one-step growth curve of HH109 is from 10 to 160 mpi, which includes three periods: a latent period (divided into an eclipse phase and an intracellular accumulation phase), a lysis period, and a stable period. Red dotted arrows indicate the time points of sample collection. Data are displayed as the means ± SD from three independent experiments.

Phage HH109 prevented most growth of V. alginolyticus culture even with a multiplicity of infection (MOI) of 1:1,000 (Fig. 1A). An MOI of 0.01 was selected for subsequent analyses. A titer assay indicated that approximately 80% of phage HH109 is adsorbed to bacterial cells within 10-min incubation (Fig. 1B). After adsorption, there was a latent period of approximately 30 min during which the phage titer in the supernatant remained unchanged. The lysis period then began before moving into a stable period where there were approximately 32.5 PFU per infected cell (Fig. 1C).

**FIG 2:**
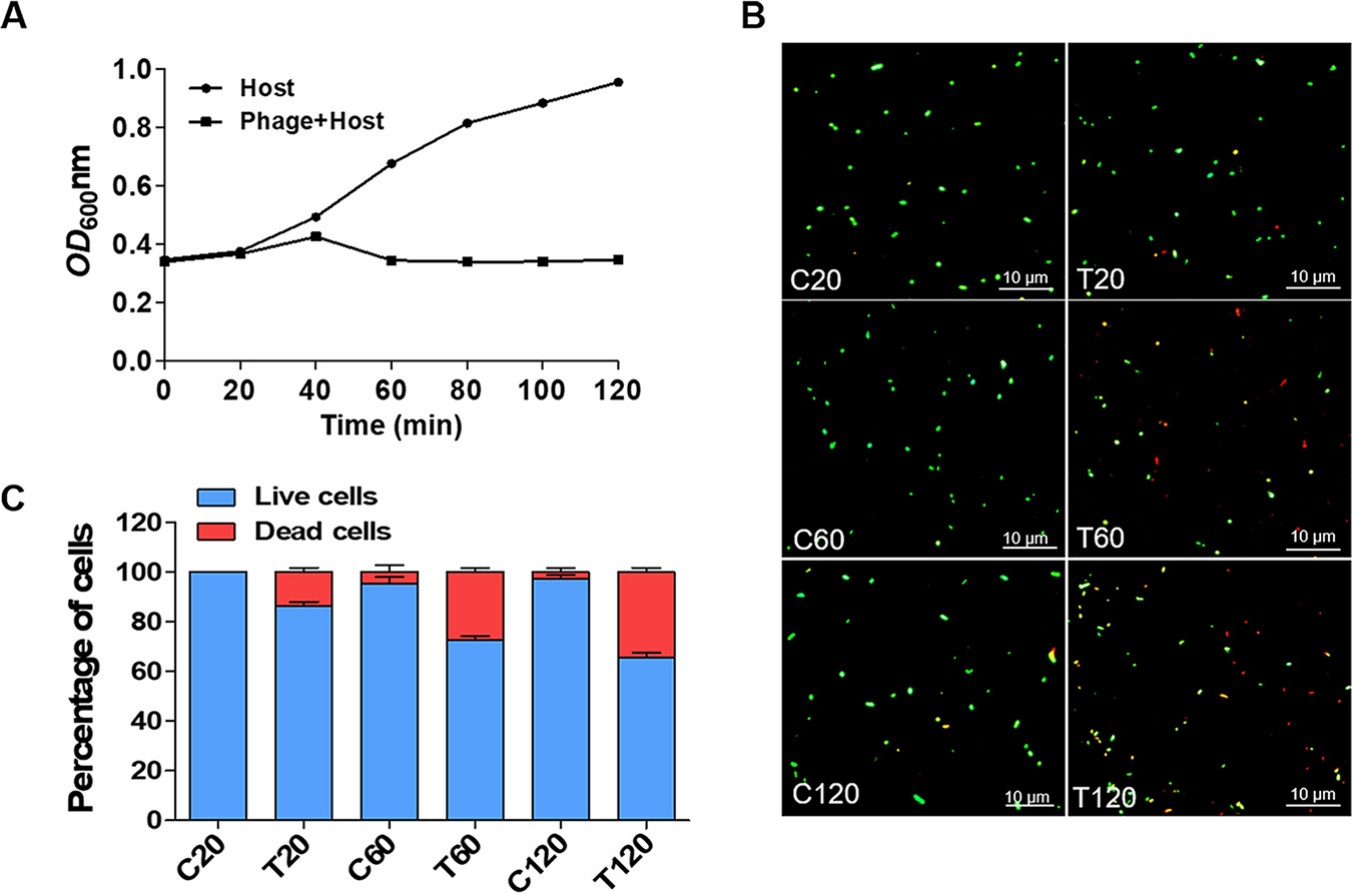
Viability of V. alginolyticus E110 for the transcriptional analysis. (A) Lysis was monitored by measuring the OD_600_ values for samples taken at the indicated times after infection. (B) Fluorescent staining: green means live bacteria, red means dead bacteria, yellow means live and dead bacteria. (C) Percentage of live and dead bacteria. Data are displayed as the means ± SD from three independent experiments.

The turbidity (OD_600_) of HH109-infected and uninfected cultures increased at almost equal rates, suggesting that phage HH109 did not affect the growth rate of the V. alginolyticus culture with the first 20 min of infection (Fig. 2A), consistent with 86.1% of cells staining as live for this time point (Fig. 2B and C). The OD_600_ of phage-treated cultures at 60 and 120 min showed limited increase in turbidity (Fig. 2A), but progressive loss of viable cells although the change was not great between 60 and 120 min (Fig. 2B and C). Based on these findings, we elected to generate transcriptional profiles for 20-, 60-, and 120-min postinfection.

### Temporal patterns of HH109 transcription.

**FIG 3:**
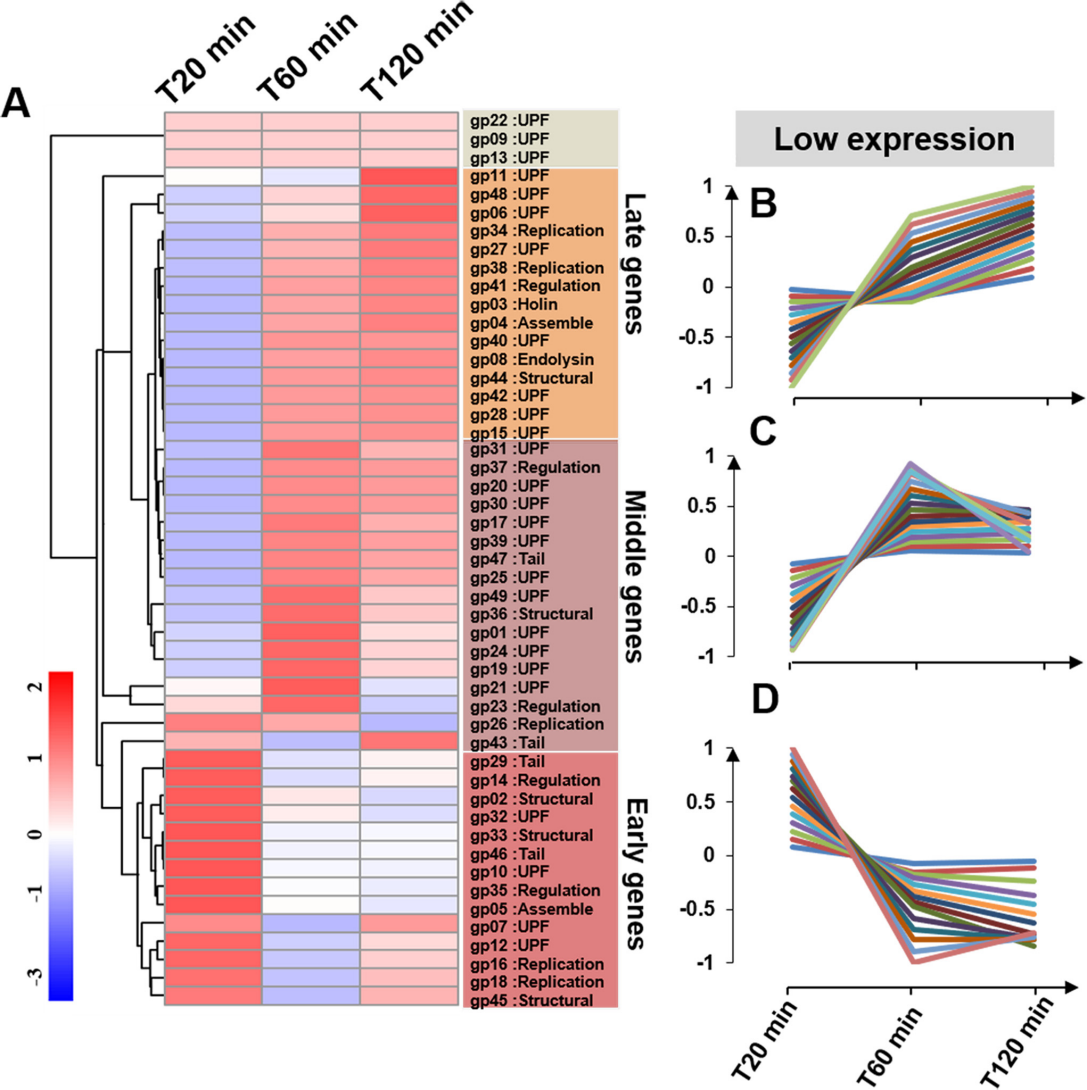
Temporal kinetic transcriptional profile of HH109 genome. (A) Hierarchical cluster heat map of HH109 genes. The normalized expression levels across all the time points are color-coded: red for high- and green for low-expression levels. Based on the read counts of genes, hierarchical cluster analysis was performed using ward.D2 and Minkowski methods (73). (B to D) A total of 49 ORFs of HH109 genes (*gp01* to *gp49*) were clustered into three clusters: early, middle, and late genes, also showing the time course of three transcript classes. The *y* axis showed the normalized expression levels of each gene. The positive sign represented a higher expression level, while the negative sign represented a lower expression level.

Transcriptional analysis revealed distinctive patterns of expressions of the 49 phage genes that included four groupings: (i) 14 ORFs peaked at 20 min (early); (ii) 17 ORFs at 60 min (middle); (iii) 15 ORFs peaked at 120 min (late); and (iv) three ORFs exhibited overall low-expression levels (*gp09*, *gp22*, and *gp13*) (Fig. 3). Some of the early genes, such as *gp46* and *gp45*, were annotated as important to host adsorption, which corresponds well with our findings. Most middle genes involved nucleotide metabolism-associated genes that are involved in phage genome replication and recombination/repair, including a DNA-directed DNA polymerase (*gp26*), a hydrolase/topoisomerase-primase (*gp23*), and a phosphomevalonate kinase (*gp37*). Late-expressed genes were mostly related to phage structural and lysis genes, like scaffolding protein (*gp44*), small terminase subunit (*gp04*), endolysin (*gp08*), and holin (*gp03*). The three hypothetical ORFs with a low relative expression were annotated as unknown proteins. Comparing the temporal transcriptional map of phage YerA41 and JD032, *gp08* and *gp03* with high similarity were expressed in the same phase, confirming our classification of HH109 genes (24, 25).

### HH109 infection induces more host gene expression in the middle and late stages.

The complete genome of strain E110 was sequenced for subsequent analysis of host-phage interactions using the Novaseq platform (GenBank: SRX10810502). The high-quality reads represented 99% of the raw reads, and high-quality sequence bases represented 99% of the raw data. The CheckM assessment and classification showed the genome assembly of E110 was 100% complete with 0.05% contaminated, ensuring a critical foundation for the inference of host-phage interactions (Table S2). It is worth mentioning that the genome contaminations reported by CheckM was due to presence of duplications of single-copy marker genes, which might represent the presence of paralogs in the E110 genome.

10.1128/msystems.00106-22.2 TABLE S2 Phylogenomic sequence alignments. Download Table S2, XLSX file, 0.01 MB. Copyright © 2022 Li et al. 2022 Li et al. https://creativecommons.org/licenses/by/4.0/ This content is distributed under the terms of the Creative Commons Attribution 4.0 International license.

**FIG 4:**
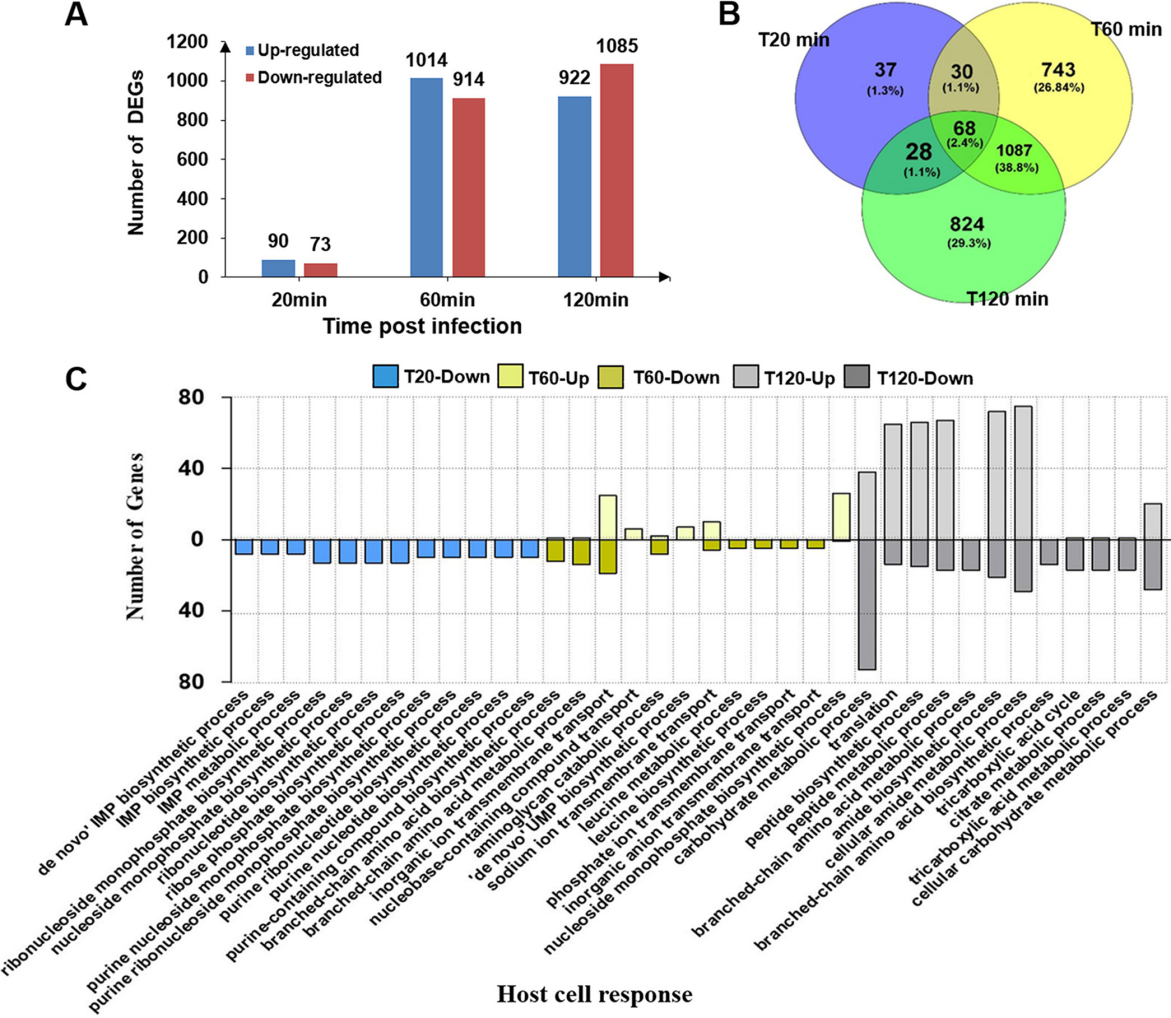
Analysis of differentially expressed genes (DEGs) of V. alginolyticus induced by phage infection. (A) The number and distribution of DEGs in different infection stages. (B) The Venn diagram shows the intersection of the number of DEGs at each time point. (C) GO analysis for the host cell response (the number of up- and downregulated genes). Upregulated genes are >0 and downregulated genes are <0.

Using the same time points as previously and an MOI of 0.01, 24.7% (1143/4620) of recognized genes were differentially expressed (DEG) for treated versus untreated cultures (16.5% upregulated genes and 18.2% downregulated genes) (Fig. 4A). Among these DEGs, 743 and 824 genes were different at 60- and 120-min postinfection, respectively, while 37 genes were differentially expressed at 20-min postinfection (Fig. 4B). During early-stage infection, bacterial genes involved with nucleic acid biosynthesis were upregulated including ribonucleoside-diphosphate reductase subunit alpha (*nrdA*) and subunit beta (*nrdB*), single-stranded DNA-binding protein (*ssbB*), DNA helicase IV (*helD*), and putative transcriptional regulator (TetR/AcrR family) (*ycnC*). At the middle stage, endoribonuclease (*ybeY*), ribose ABC transporter ribose-binding lipoprotein (*rbsB*), oligopeptide transporter permease (*poT*), ribose ABC transporter permease (*rbsC*), a membrane transport protein (*rbsD*), TonB-dependent siderophore receptor (*tbrS*), ABC transporter ATP-binding protein (*rbsA*), and major facilitator transporter (*mfS*) genes were downregulated, and mainly involved in minor biosynthetic processes and transport. During the stable stage, DEGs included elongation factor G (*ef-G*), HAD family phosphatase (*serB*), malate dehydrogenase (*mdH*), fumarate hydratase (*FH*), glycogen synthase (*GS*), succinate dehydrogenase (*sdH)*, putative 3-hydroxyisobutyrate dehydrogenase (*hibadH*) which are mainly associated with metabolism, such as leucine metabolic process, cellular amide metabolic process, branched-chain amino acid metabolic process, citrate metabolic process, etc. That is, V. alginolyticus DEGs represented discrete function classifications for each of the three stages (Fig. 4C). The detailed expression data on DEGs are presented in Table S3.

10.1128/msystems.00106-22.3 TABLE S3 Detailed GO expression data on specific genes of V. alginolyticus. Download Table S3, DOCX file, 0.03 MB. Copyright © 2022 Li et al. 2022 Li et al. https://creativecommons.org/licenses/by/4.0/ This content is distributed under the terms of the Creative Commons Attribution 4.0 International license.

**FIG 5:**
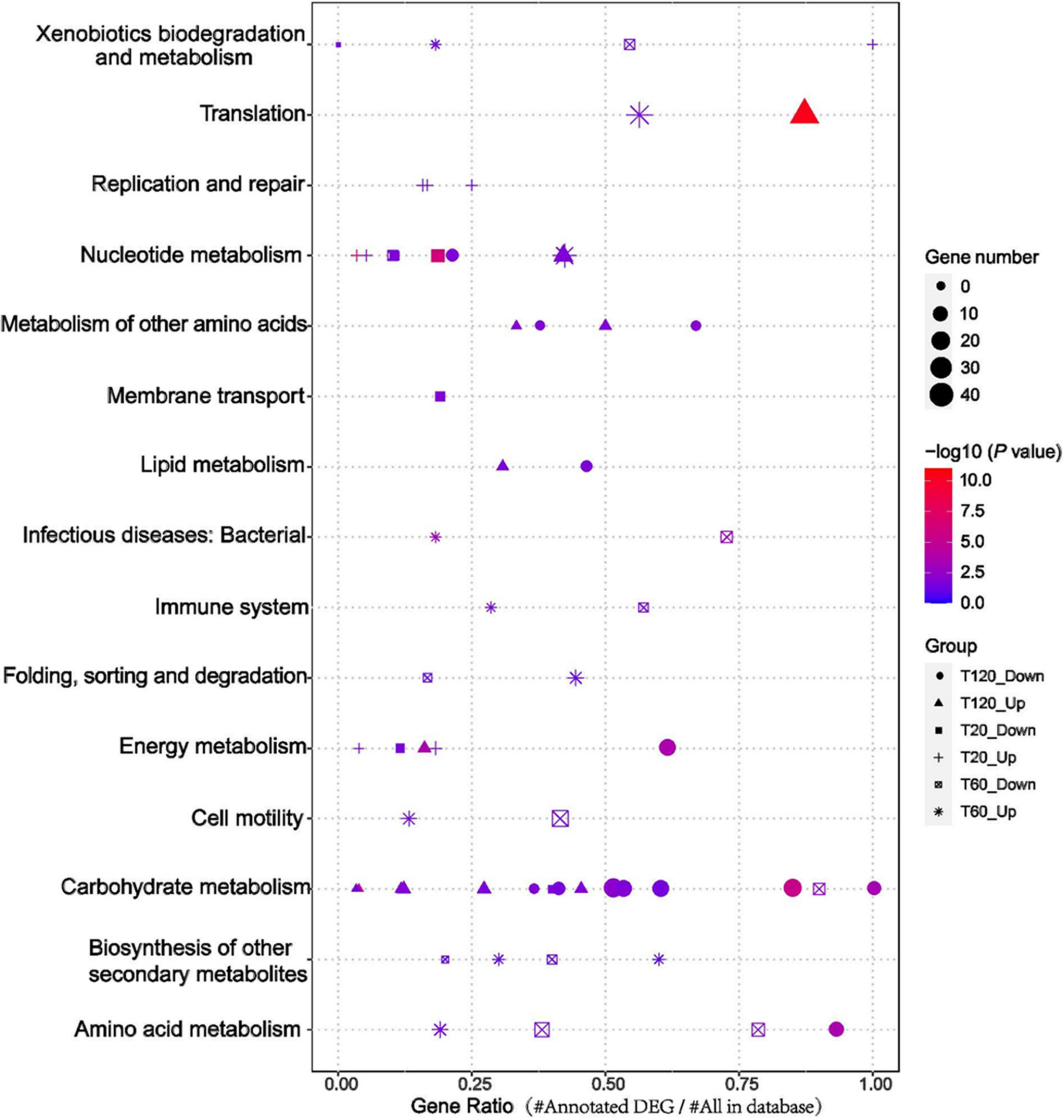
KEGG enrichment analysis of host DEGs (up- and downregulated genes) at each time point after HH109 infection. The shape of the point indicates the time points. The enrichment *P* value of each pathway was normalized as a negative log (*P* value) and is shown as a color gradient. The number of genes enriched in each pathway is represented by the size of the points. The ordinate is the KEGG pathway categories, the abscissa is the gene ratio and was calculated as number of DGEs annotated in a given KEGG pathway/total number of genes in a given KEGG pathway.

To integrate complementary information from different databases and gain insight into the functions of DEGs, a Kyoto Encyclopedia of Genes and Genomes (KEGG) pathways analysis was also carried out. Based on the adjusted *P* value (*q*) and gene number (GN) of each KEGG pathway, 15 pathways were significantly changed (*q* <0.05 and GN > 3) at one time point or more. From the time distribution of pathways, the DEGs mainly involved the host’s nucleotide replication and metabolisms, such as DNA replication and pyrimidine metabolism in the early stage of infection. The most extensively changed KEGG pathways occurred in the middle and late stages of infection, which involved metabolites, such as carbohydrate metabolism, energy metabolic, lipid metabolic, *etc.* (Fig. 5). The detailed expression data on pathways are presented in Table S4.

10.1128/msystems.00106-22.4 TABLE S4 Detailed KEGG pathways expression data on specific genes of V. alginolyticus. Download Table S4, DOCX file, 0.03 MB. Copyright © 2022 Li et al. 2022 Li et al. https://creativecommons.org/licenses/by/4.0/ This content is distributed under the terms of the Creative Commons Attribution 4.0 International license.

### Effects of HH109 infection on host V. alginolyticus drug resistance and virulence genes.

**FIG 6:**
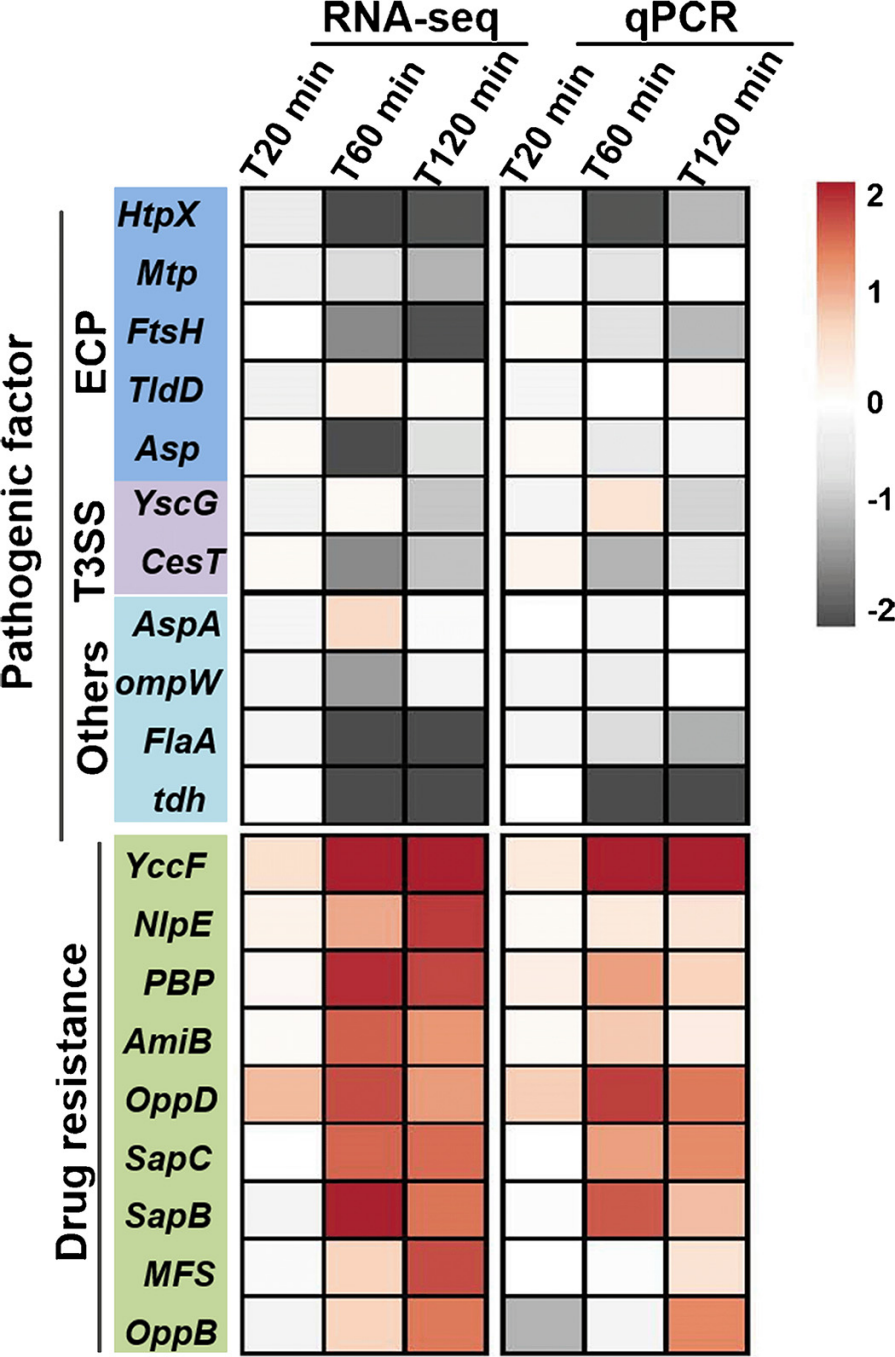
Expression of host genes related to pathogenicity and drug resistance after phage infection. Colors indicate upregulation and downregulation of the genes. ECP, extracellular products. T3SS, genes associated with type III secretion system of host.

We assessed the effects of phage HH109 infection on expression of 11 putative virulence genes and nine antibacterial resistance genes using both RNA-seq and qPCR. The results showed that most virulence-associated genes in V. alginolyticus were severely inhibited. In contrast, the antimicrobial resistance genes were mostly upregulated after the early stage (Fig. 6). The upregulation of these resistance-related genes or downregulation of these virulence-related genes indicated their potential participation in anti-phage infection in the host.

### Phage-host interaction network.

**FIG 7:**
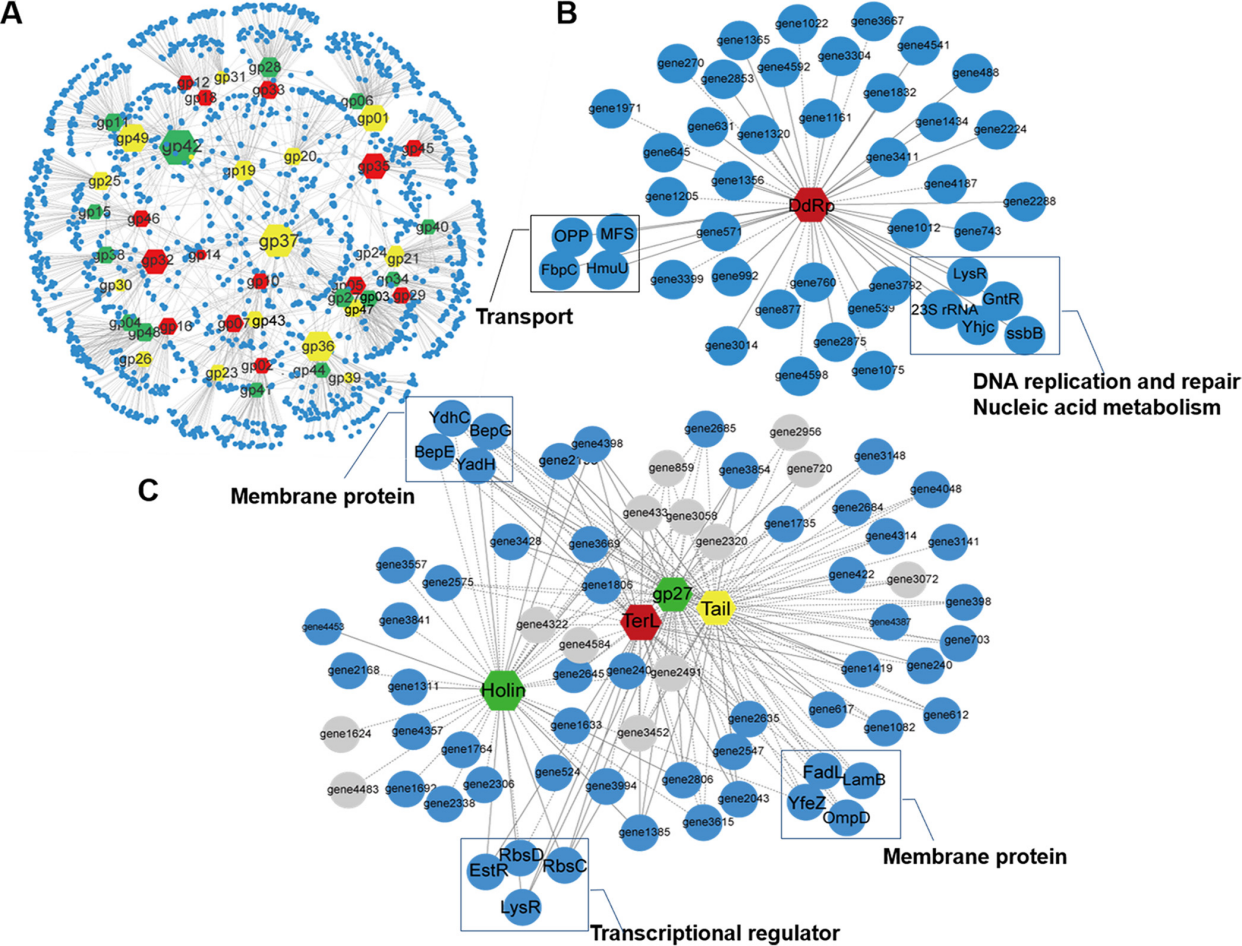
Gene co-expression network between HH109 and the host. (A) Main networks of host interactions centered on phage genes. (B) Positive and negative regulatory networks of early phage gene *DdRp*. (C) Positive and negative regulatory networks of phage gene *gp03* (Holin), *gp05* (TerL), *gp47* (tail protein), and *gp27* (hypothetical protein). The hexagons represent phage genes, three colors of nodes represent the early (red), middle (yellow), and late (green) HH109 genes, respectively; the blue and gray nodes are the annotated or hypothetical genes of host bacteria, respectively. The size of the nodes shows the interaction strength.

To assess potential interactions between phage and host gene expression, we constructed interaction networks (Fig. 7). Based on the sequential expression of the HH109 phage genes, classified into early, middle, late, and low-expression genes (Fig. 3A), we found a total of 2,275 co-expression relationships (*P < *0.01, |*r*| > 0.99), including 436 negative correlations and 1,839 positive correlations (*P < *0.01, *r *> 0) (Table S5). The correlation between HH109 and V. alginolyticus genes was quantified by a K-score range from one to five. Genes with higher K-scores (more links) were shown in bigger node sizes denoting their potential regulatory roles (Fig. 7). Large nodes included HH109 early genes *gp32* (uncharacterized protein), *gp35* (calcineurin-like phosphoesterase), middle genes *gp01* (viron protein), *gp36* (uncharacterized protein), *gp49* (uncharacterized protein), *gp37* (phosphomevalonate kinase), and late gene *gp42* (uncharacterized protein) (Fig. 7A). Sub-network linkages show genes with similar biological functions (26) such as the linkage between *gp16* (DNA-directed RNA polymerase, DdRp) and five host genes (transcriptional regulator, *gntR*, 23s rRNA, *lysR*, etc.) related to DNA replication and repair, nucleic acid metabolism (Fig. 7B). Genes *gp03* (Holin), *gp05* (large terminase subunit, TerL), *gp47* (tail protein), and *gp27* (hypothetical protein) composed one sub-network that could be defined as a transcription and membrane protein network (Fig. 7C). The inclusion of *gp27* suggests that it contributes to this function and further comparisons with the UniProt database indicated some amino acid similarity to ORF68 (DNA-directed RNA polymerase I subunit RPA49; 53.3% similarity) and DNA helicase from Pontibacillus marinus (36.4% similarity).

10.1128/msystems.00106-22.5 TABLE S5 Co-expression data of phage HH109 genes and host DEGs. Download Table S5, XLSX file, 1.1 MB. Copyright © 2022 Li et al. 2022 Li et al. https://creativecommons.org/licenses/by/4.0/ This content is distributed under the terms of the Creative Commons Attribution 4.0 International license.

### Experimental validation of selected DEGs by qPCR.

**FIG 8:**
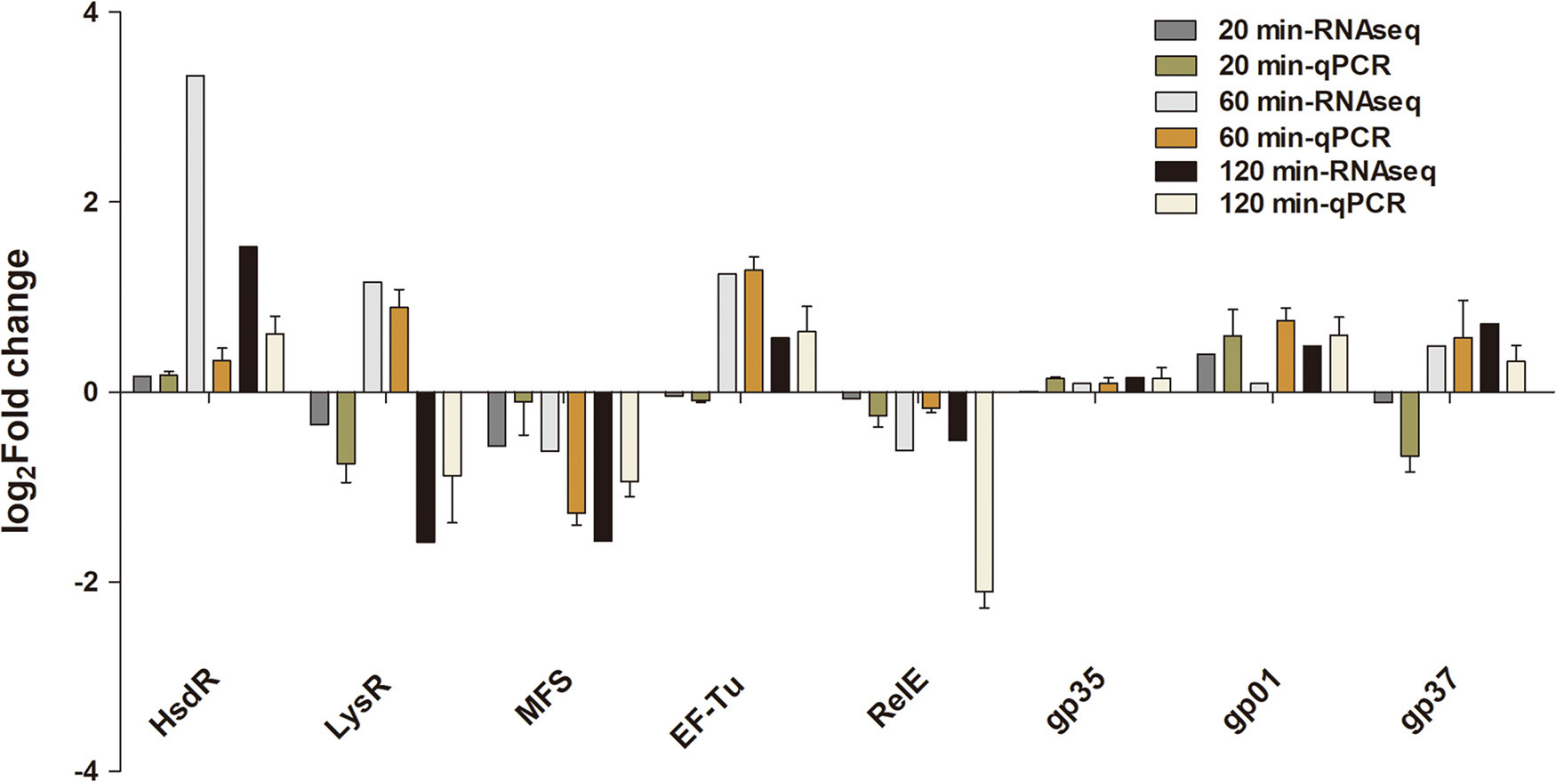
RT-qPCR validation of selected DEGs. Eight genes from V. alginolyticus and HH109 were compared by both qRT-PCR and RNA-seq. The qPCR results were normalized using *DnaK* and expressed as fold change (Log_10_ scale) by the comparative Ct method; moreover, the overall correlation coefficient for these pairwise comparisons were 0.87 (*n* = 24, *P < *0.0001).

We used qRT-PCR to validate the results of RNA-seq for three phage genes (*gp01*, *gp35*, *gp37*) and seven bacterial genes (*hsdR*, *lysR*, *mfS*, *ef-tu*, *relE*) (Fig. 8). Comparative analysis showed that the expression pattern detected by qRT-PCR was a consistent directional change compared with RNA-seq, and change compared to RNA-seq with an overall correlation coefficient (*r*) of 0.87 (*n* = 24, *P < *0.0001). These results are consistent with robust findings from the RNA-seq analysis.

## DISCUSSION

The study of host-phage relationships is essential to understanding the mechanism of phage infection, coevolution of phages and hosts, as well as the complexity and diversity of phage-bacterium interactions (27). However, current knowledge about phage-host interactions is mainly based on a small number of bacterial hosts, including E. coli, Acinetobacter baumannii, P. aeruginosa (21, 28), and few studies consider both phage and host responses (28, 29). Therefore, probing the viral effects in a different but well-known bacterium, like V. alginolyticus, is essential for a deeper understanding of the interaction mechanism between host-phage. To this end, the temporal transcriptomic dynamic interactions between phage HH109 and its host V. alginolyticus were investigated using time-resolved RNA-seq. Our results demonstrated that the expression of HH109 genes follows the standard lytic phage pattern, including early, middle, and late stages. Transcriptional responses of V. alginolyticus to the phage HH109 infection were time-dependent, and presumably, many bacterial resources were needed for phage HH109 assembly and release in the middle and late infection stages. Moreover, the gene-gene interaction networks between HH109 and the host V. alginolyticus reflected probable protein-protein interactions between HH109 and host cells.

Previous work showed that lytic phages φSt2 and φGrn1 released progeny virions from V. alginolyticus after 20 min of infection, reaching the maximum at approximately 60 min when the infective cycle was completed (30). The latent period for the V. alginolyticus phage A318 was 20 min, followed by a 20-min rise period and an average burst size of approximately 72 phage particles per infected cell (31). In the present study, we found that the propagation cycle of phage HH109 was 100 min and the lysis period appeared at 40 to 100 min; moreover, progeny phage had not been released in the first 40 min (Fig. 1C). Compared with lytic phage VEN, these results suggested that the lytic cycle of HH109 is longer compared with other lytic *Vibrio* phages (32).

Adsorption is the first step in phage infection (33). Tail proteins serve as an adsorption organ for phage, and a series of subsequent infections are initiated when binding to receptors on the host cell surface (34). Like the tail protein, major capsid proteins adsorb to the pilus of E. coli and this initiates a cascade of processes involved in lysing E. coli (35). For the current study, tail protein (*gp46*) and major capsid protein (*gp45*) expression peaked during the early expression phase (Fig. 3). Endolysin and holin are involved with termination of the growth cycle and release viral progeny through host cell lysis (36), which is consistent with peak expression of these proteins during the late stage (Fig. 3).

The timing of the lytic cycle can vary between different phage. For instance, *Myoviridae* phage JD007 inhibited the growth of Staphylococcus aureus after coculture for 30 min at an MOI >1 (37). In contrast, *Podoviridae* phages VEN did not affect the growth rate of V. alginolyticus culture with MOI 10 within approximately an hour (32). For the current study, host cell genes involved with growth and division were not affected during the first 20 mpi (Fig. 4C and Fig. 5), consistent with different kinetics of lysis and phage-host genetic interactions. Others have shown that phage infection leads to specific protein-protein interactions whereby phage proteins inhibit, activate or otherwise redirect specific host proteins (38, 39) and this is presumably the case for phage HH109 as well with the exception that we observed far more upregulated proteins compared with downregulated proteins, which is different from other studies (21, 28).

DEGs from the V. alginolyticus host could be broadly grouped as involving nucleic acid biosynthesis in the early infection phase, transport in middle phase, and metabolic processes in stable infection stages. For example, early phase upregulation included *nrdA* and *nrdB* host genes that encode ribonucleoside diphosphate reductase in phage T4, which is the key enzyme in the synthesis of DNA and catalyzes the first irreversible, committed step in the synthesis of dNTPs from rNDPs (40). YcnC is a putative transcriptional regulator of the TetR/AcrR family. These proteins are essential for the biosynthesis of deoxyribonucleotides from the corresponding ribonucleotides (41). Single-stranded DNA binding proteins (*ssbB*) are essential to the cell as they stabilize transiently open single-stranded DNA (*ssDNA*) intermediates, recruit appropriate DNA metabolism proteins, and coordinate fundamental processes such as replication, repair, and recombination (42). In addition, SsbB from Bacillus subtilis could contribute to some extent to stimulate replication of viral DNA (43). Furthermore, HelD is a helicase implicated in DNA repair and homologous recombination and interact with the RNA polymerase to stimulate effect on transcription cycling and elongation (44).

After 60 min of infection (middle phase), there is an induction of *YbeY*, which is involved in the processing of all three rRNAs and its products (RNase III, RNase R, and PNPase) that play important roles in both rRNA maturation and RNA degradation in E. coli (45), and thus *ybeY*, could have an impact in translation (46). Bacterial ABC transporters are involved in several diverse processes, including multidrug resistance, protein secretion, quorum sensing, and nutrient uptake (47). The members of SBP ABC transporters are located in the periplasm of Gram-negative bacteria and can accumulate solutes against large concentration gradients (48). In addition, genes *rbsA*, *rbsB*, *rbsC*, and *rbsD* constitute a ribose transport operon to provide a resource for carbon and energy synthesis (49). Therefore, the most extensively changed functions and pathways were seen at 60 min and involved the processes of transport and minor biosynthetic pathways in the present study.

Previous studies have not investigated changes in host gene expression during the stable phase following phage infection. For the present study, the majority of host cells were lysed by 120-min postinfection, leaving only small numbers of uninfected host (presumably phage HH109 resistant strains) or cellular debris (21). The host V. alginolyticus response to phage HH109 infection during the stable phase was markedly different. For example, there were more bacterial DEGs at the 120-min time point than those during other periods (Fig. 4A). EF-G is a translation GTPase, which catalyzes tRNA movement during translation elongation, and promotes ribosomal recycling in the last step of translation. Moreover, EF-G is involved in various metabolic processes such as branched-chain amino acid metabolism (50). SerB proteins belong to the HAD phosphatase family, a relatively less-studied enzyme family that is involved in various metabolic processes including cellular-, phosphorus-, and phosphate-containing compound metabolism (51). SDH is a mitochondrial enzyme that participates in both the citric acid cycle and the electron transport chain. The SDH substrate succinate plays an important role in metabolic processes (52). Analysis of putative protein function indicated that DEGs of the host V. alginolyticus were significantly different from the other periods, mainly involving metabolism, especially carbohydrate metabolism in the present work (Fig. 4C and Fig. 5).

Like many other opportunistic pathogens, V. alginolyticus utilizes a variety of carbohydrates as sources of carbon and energy, and carbohydrates serve functions in combating host defenses and phage invasion (53). In addition, carbon sources, such as glucose, provide energy for the rapid growth of host bacteria and play a critical role in controlling cell death (54). Clustering of KEGG pathways revealed that carbohydrate metabolism was significantly inhibited during the infection period (Fig. 5), which may be important for keeping the host in a physiological state necessary for optimal phage development.

Previous reports have shown that prophages can promote the expression and induction of virulence/resistance traits when infecting Enterococcus faecalis, P. aeruginosa, E. coli, and Bacillus anthracis (55–58). For the current study, infection with the lytic phage HH109 was associated with downregulation of virulence-associated genes and upregulation of drug-resistance genes.

Gene co-expression network analysis has the potential to identify putative gene function (59). For example, using gene co-expression networks, Yang reported that a middle gene *gp34* of phage Abp1 had a negative interaction with numerous host ribosome protein genes (28), and the hypothetical proteins ORF68 and ORF59 of phage PaP3 might contribute to control of energy metabolism (21). In the present study, gene *gp27* of phage HH109 is included with Holin (*gp03*), large terminase subunit (*gp05*), and tail protein (*gp47*) that are involved in membrane transport and transcriptional regulation. Consequently, the uncharacterized gene *gp27* is likely to be involved these functions as well.

One potentially important weakness for this study involved the loss of approximately 25% of cells in the middle and stable phase samples due to lysis. It possible that this influenced the results through reduced assay sensitivity, or through genetic effects triggered by the presence of dead cells and lysate rather than due to direct effects of the phage.

In conclusion, global transcriptional analysis of phage HH109 and its host V. alginolyticus highlighted stage-dependent inhibition of host genes by HH109 and suggested that the late gene *gp27* of phage HH109 is involved in membrane transport and/or transcriptional regulation, and there is elevated gene expression associated with antibiotic resistance during lytic infection. Further, phage-mediated suppression of carbon resource associated genes from infected cells might indicate suppression of host defense mechanisms. Future functional studies (e.g., gene knockout, complementation, and heterologous expression) will provide more direct evidence of how phage and host genes and their products are involved during phage infection.

## MATERIALS AND METHODS

### Bacteria and phage isolation and preparation.

V. alginolyticus strain E110 was originally isolated from shrimp in a South China aquaculture farm (23). Bacteria were cultured in LB liquid medium with 2% NaCl at 37°C. The phage HH109 was isolated using the V. alginolyticus strain E110 as host and purified using the standard double-layer agar method (30). The phage HH109 (3.8 × 10^10^ CFU/mL) was stored in Tris-SM buffer at 4°C.

### Determination of the optimal multiplicity of infection (MOI).

Aliquots of bacterial cells (2.4 × 10^8^ CFU/mL) were infected with phage HH109 at MOI of 0.001, 0.01, 0.1, or 1, respectively. Subsequently, the cocultures were grown at 37°C with continuous shaking (180 rpm), and the optical density (OD) OD_600_ values were measured to evaluate the growth rate of bacterial cells using the Bioscreen C (Growth Curves Ab Ltd) at every 20-min interval over a 160-min time course.

### Adsorption curve and one-step growth curve.

The adsorption rate of phage HH109 on V. alginolyticus was determined in a previously described method (31). Briefly, bacterial cells were grown in 10 mL of LB broth with 2% NaCl to a density of 10^6^ CFU/mL and then infected with the phage HH109 at MOI of 0.01 over a 10-min time course. The mixtures were collected at the indicated time point and pelleted at 14,000 × *g* for 30 s. Subsequently, the phage titer in the supernatant was measured by the double-layer agar plate method (31).

To ascertain the infection dynamics of phage HH109, the one-step growth curve of the phage was defined according to the method described previously (60). In brief, 20 mL of bacterial cells with a density of 10^6^ CFU/mL were infected with phage HH109 at MOI of 0.01. After 10 min of adsorption, the mixtures were centrifuged at 14,000 × *g* for 1 min to remove the supernatant containing un-adsorbed phages. The pellets were washed twice with phosphate-buffered saline (PBS) and resuspended in 20 mL of fresh LB broth with 2% NaCl, then incubated at 37°C with shaking at 180 rpm. Samples (0.5 mL) were successively removed at indicated times over a 160-min period. Supernatants were obtained after centrifugation for 30 s at 14,000 × *g*, and the phage titer was immediately measured using the double-layer agar plaque method.

### Whole-genome sequencing of V. alginolyticus strain E110.

The genomic DNA of the V. alginolyticus strain E110 was extracted using a Puregene Yeast/Bact. kit B (Qiagen, Germany). The DNA samples’ concentration, quality, and integrity were evaluated using Quant-iT PicoGreen dsDNA assay kit and 1% agarose gel electrophoresis, respectively. DNA Libraries were constructed with Illumina TruSeq DNA sample preparation reagents according to the manufacturer’s instructions and sequenced using an Illumina NovaSeq instrument (Illumina, United States) owned by Shanghai Personal Biotechnology Co., Ltd. Adapter Removal was employed to remove linker sequences (61). The post-trimming high-quality reads were used to generate the genome assembly integrating A5-MiSeq v20150522 (62), SPAdes (63), pilon (64), respectively. The quality, completeness, and contamination of the V. alginolyticus genome were assessed using CheckM (65). The prokaryotic gene model was predicted by GeneMarkS (66) and annotated BLAST plus (67) using the NCBI-nr database.

### Bacteria-phage interactions resolved by RNA-seq time-series.

Based on the adsorption curve and one-step growth curve, 20 mL cultures of V. alginolyticus E110 were collected at 20, 60, and 120 min after phage HH109 infection at MOI of 0.01 and pelleted by centrifugation at 14,000 × *g* for 2 min. In parallel, uninfected E110 cells with equal volume and identical time points were collected as uninfected controls. All samples were immediately frozen at −80°C before RNA extraction. In addition, an aliquot of culture was taken at the three times to monitor the V. alginolyticus viability by optical density and fluorescence live/dead staining according to the manufacturer’s instructions (Thermo Scientific, United States).

Total RNA was extracted from each sample using TRIzol reagent according to the manufacturer’s instructions (Invitrogen Life Technologies). The concentration, quality, and integrity of the RNA samples were determined using NanoDrop ND-1000 UV spectroscopy (Thermo Scientific, United States) and 2100 Bioanalyzer (Agilent Technologies, United States) with RNA 6000 Nano kit (Walvax Biotechnology Co., Ltd.), respectively. The RNA samples with RNA integrity values (RIN) ≥ 8 were selected to construct cDNA libraries. Post-quality-control RNA samples were further subjected to mRNA enrichment by depleting rRNA with RiboZero magnetic kit (Bacteria; MRZMB126) supplied by Illumina. Random oligonucleotides and SuperScript III were used to synthesize the first strand cDNA. RNaseH was then used to degrade the RNA strand, and DNA polymerase I was used with dNTP and dUTP instead of dTTP to generate the second strand of cDNA. Remaining overhangs were converted into blunt ends via exonuclease/polymerase activity and the enzymes were removed by NEBNext Ultra Directional RNA Library Prep Kit for Illumina. After adenylation of the 3′ ends of the DNA fragments, Illumina PE adapter oligonucleotides were ligated to prepare for hybridization. cDNA library fragments were purified with the AMPure XP system (Beckman Coulter, Beverly, USA) to ensure preferential 400 to 500 bp length. The PCR cycles were adjusted to 15, and the final amplified libraries were quality checked using Bioanalyzer 2100 system (Agilent). Finally, quantitative PCR was used with the Kapa-SYBR FAST qPCR kit for Light Cycler 480 (KK4610) from Kapa Biosystems and a reference standard to construct equimolar pool of libraries. Each library was sequenced using TruSeq SBS kit v3-HS, in paired-end mode with the read length of 2 × 76 bp for the mRNA-seq experiments, using the HiSeq2000 instrument (Illumina) according to the manufacturer’s protocol.

### Bioinformatics analysis of RNA-seq data.

FastQC was used to evaluate the quality of RNA sequencing reads. Raw data in the fastq format were preprocessed using sickle (version 1.2) (68). Clean data were obtained by removing adapter sequences, poly-N, and low-quality reads from raw data. All the downstream analyses were based on clean data with high-quality determined by Q30. Differential gene and transcript expression values were calculated according to the expected number of fragments per fragments per kilobase of exon model per million mapped fragments (FPKM). DESeq (v1.30.0) was used to detect differentially expressed mRNA, defined as transcripts with |log_2_FoldChange|>1 and *P*-value < 0.05. *P*-values were calculated by using a negative binomial distribution and corrected for compounding error by Benjamini-Hochberg (B & H). V. alginolyticus genes with a fold change value (FC) of ≥1.5 and a *q* value of ≤ 0.05 after phage HH109 infection were analyzed and classified. gene ontology (GO) analysis was performed with Blast2GO (BioBam) based on Wallenius’ non-central hypergeometric distribution (http://bowtie-bio.sourceforge.net/index.shtml). KOBAS was used for the KEGG pathway analysis (69). Gene co-expression networks were developed by using Cytoscape 3.4.0 (70). Circos was used to draw the graph of the merged network for co-expressed genes (71).

### RT-qPCR validation of RNA-seq results.

To validate the RNA-seq data, 20 virulence- or drug resistance-related genes of host V. alginolyticus, five phage-bacteria interaction genes, and three HH109 genes were selected for qRT-PCR analysis. Primers were designed using Primer Premier 5 software (Table S1). RNA (1 μg) was reverse transcribed into cDNA using the PrimeScript RT reagent kit (TaKaRa, Japan) following the manufacturer’s instructions with random primers. The PCR product was sequenced to ensure specificity of the amplified fragments. *DnaK* rRNA was used as an internal control to normalize the expression level, and all experiments were performed in triplicate. The qRT-PCR system was carried out in a 20-μL volume containing 0.4 μL one each of primer set (10 μM), 10 μL 2×SuperMix (Thermo Fisher Scientific), 8.2 μL ddH_2_O, and 1 μL diluted cDNA template. The amplification protocol was set as follows: 94°C for 5 min, 40 cycles of 10 s at 94°C, 30 s at 60°C. Relative expression levels of target genes were calculated using the comparative Ct method with formula 2^−△△Ct^ (72).

10.1128/msystems.00106-22.1 TABLE S1 Details of the primer sequence used for qPCR. Download Table S1, DOCX file, 0.02 MB. Copyright © 2022 Li et al. 2022 Li et al. https://creativecommons.org/licenses/by/4.0/ This content is distributed under the terms of the Creative Commons Attribution 4.0 International license.

### Data availability.

All the raw sequencing data generated from this study including DNA and RNA-seq are publicly available at the NCBI database under accession number PRJNA727447.
