# Supplementary material for: Temporal Transcriptional Responses of a Vibrio alginolyticus Strain to Podoviridae Phage HH109 Revealed by RNA-Seq
Source: mSystems. 2022 Apr 11;7(2):e00106-22. doi: 10.1128/msystems.00106-22 (PMC9040624; doi:10.1128/msystems.00106-22)
Supplement: TABLE S3 [file msystems.00106-22-s0003.docx]

**Table S3**

**Detailed GO expression data on specific genes of *V. alginolyticus***

| **GO_accession** | **Description** | **Group** | ***P-*value** | **FDR** | **Gene_list** |
| --- | --- | --- | --- | --- | --- |
| GO:0006189 | de novo' IMP biosynthetic process | 20 min Down | 5.47E-10 | 5.119E-07 | gene1207, gene1240, gene2773, gene3124, gene3547, gene4080, gene4081, gene4488 |
| GO:0006188 | IMP biosynthetic process | 20 min Down | 1.59E-09 | 5.119E-07 | gene1207, gene1240, gene2773, gene3124, gene3547, gene4080, gene4081, gene4488 |
| GO:0046040 | IMP metabolic process | 20 min Down | 1.59E-09 | 5.119E-07 | gene1207, gene1240, gene2773, gene3124, gene3547, gene4080, gene4081, gene4488 |
| GO:0009156 | ribonucleoside monophosphate biosynthetic process | 20 min Down | 3.743E-09 | 9.04E-07 | gene1207, gene1240, gene1728, gene2337, gene2773, gene3124, gene3502, gene3503, gene3547, gene3939, gene4080, gene4081, gene4488 |
| GO:0009124 | nucleoside monophosphate biosynthetic process | 20 min Down | 6.877E-09 | 1.329E-06 | gene1207, gene1240, gene1728, gene2337, gene2773, gene3124, gene3502, gene3503, gene3547, gene3939, gene4080, gene4081, gene4488 |
| GO:0009260 | ribonucleotide biosynthetic process | 20 min Down | 7.323E-08 | 1.179E-05 | gene1207, gene1240, gene1728, gene2337, gene2773, gene3124, gene3502, gene3503, gene3547, gene3939, gene4080, gene4081, gene4488 |
| GO:0046390 | ribose phosphate biosynthetic process | 20 min Down | 1.158E-07 | 1.598E-05 | gene1207, gene1240, gene1728, gene2337, gene2773, gene3124, gene3502, gene3503, gene3547, gene3939, gene4080, gene4081, gene4488 |
| GO:0009127 | purine nucleoside monophosphate biosynthetic process | 20 min Down | 2.035E-07 | 2.184E-05 | gene1207, gene1240, gene2773, gene3124, gene3502, gene3503, gene3547, gene4080, gene4081, gene4488 |
| GO:0009168 | purine ribonucleoside monophosphate biosynthetic process | 20 min Down | 2.035E-07 | 2.184E-05 | gene1207, gene1240, gene2773, gene3124, gene3502, gene3503, gene3547, gene4080, gene4081, gene4488 |
| GO:0009152 | purine ribonucleotide biosynthetic process | 20 min Down | 3.046E-06 | 0.0002942 | gene1207, gene1240, gene2773, gene3124, gene3502, gene3503, gene3547, gene4080, gene4081, gene4488 |
| GO:0006164 | purine nucleotide biosynthetic process | 20 min Down | 3.816E-06 | 0.0003351 | gene1207, gene1240, gene2773, gene3124, gene3502, gene3503, gene3547, gene4080, gene4081, gene4488 |
| GO:0072522 | purine-containing compound biosynthetic process | 20 min Down | 8.833E-06 | 0.000711 | gene1207, gene1240, gene2773, gene3124, gene3502, gene3503, gene3547, gene4080, gene4081, gene4488 |
| GO:0009082 | branched-chain amino acid biosynthetic process | 20 min Down | 0.0004252 | 0.8511059 | gene2198, gene2501, gene2502, gene2503, gene2504, gene2511, gene2512, gene2815, gene4055, gene4056, gene4057, gene4059 |
| GO:0009082 | branched-chain amino acid biosynthetic process | 60 min Up | 0.0004252 | 0.8511059 | gene2766 |
| GO:0009081 | branched-chain amino acid metabolic process | 60 min Down | 0.0010024 | 0.8511059 | gene1755, gene2198, gene2501, gene2502, gene2503, gene2504, gene2511, gene2512, gene2815, gene4055, gene4056, gene4057, gene4059, gene902 |
| GO:0009081 | branched-chain amino acid metabolic process | 60 min Up | 0.0010024 | 0.8511059 | gene2766 |
| GO:0098660 | inorganic ion transmembrane transport | 60 min Down | 0.003773 | 0.8511059 | gene103, gene1333, gene1650, gene1770, gene1794, gene1795, gene1866, gene2117, gene2563, gene2663, gene2937, gene3367, gene3462, gene3463, gene3536, gene3688, gene3734, gene4272, gene944 |
| GO:0098660 | inorganic ion transmembrane transport | 60 min Up | 0.003773 | 0.8511059 | gene1039, gene1245, gene1395, gene1479, gene1488, gene1691, gene1710, gene1775, gene1916, gene1947, gene2015, gene2059, gene2711, gene2866, gene2907, gene3261, gene3708, gene4046, gene4047, gene4048, gene4049, gene4071, gene4072, gene61, gene649 |
| GO:0015931 | nucleobase-containing compound transport | 60 min Up | 0.0051156 | 0.8511059 | gene287, gene3209, gene3261, gene3708, gene505, gene61- |
| GO:0006026 | aminoglycan catabolic process | 60 min Down | 0.0105471 | 0.8511059 | gene2077, gene2134, gene3185, gene3343, gene3505, gene3922, gene4295, gene655 |
| GO:0006026 | aminoglycan catabolic process | 60 min Up | 0.0105471 | 0.8511059 | gene2662, gene4379 |
| GO:0044205 | 'de novo' UMP biosynthetic process | 60 min Up | 0.0108131 | 0.8511059 | gene1531, gene1728, gene2337, gene2609, gene2610, gene2954, gene3939- |
| GO:0035725 | sodium ion transmembrane transport | 60 min Down | 0.0112349 | 0.8511059 | gene1333, gene1650, gene2117, gene2937, gene3367, gene3536 |
| GO:0035725 | sodium ion transmembrane transport | 60 min Up | 0.0112349 | 0.8511059 | gene1245, gene1395, gene1947, gene2059, gene2866, gene2907, gene3261, gene3708, gene61, gene649 |
| GO:0006551 | leucine metabolic process | 60 min Down | 0.0123379 | 0.8511059 | gene2501, gene2502, gene2503, gene2504, gene4057 |
| GO:0009098 | leucine biosynthetic process | 60 min Down | 0.0123379 | 0.8511059 | gene2501, gene2502, gene2503, gene2504, gene4057 |
| GO:0035435 | phosphate ion transmembrane transport | 60 min Down | 0.0123379 | 0.8511059 | gene1794, gene1795, gene2663, gene3462, gene3463 |
| GO:0098661 | inorganic anion transmembrane transport | 60 min Down | 0.0123379 | 0.8511059 | gene1794, gene1795, gene2663, gene3462, gene3463 |
| GO:0009124 | nucleoside monophosphate biosynthetic process | 60 min Down | 0.0135909 | 0.8511059 | gene1866 |
| GO:0009124 | nucleoside monophosphate biosynthetic process | 60 min Up | 0.0135909 | 0.8511059 | gene1089, gene1108, gene1207, gene1531, gene1728, gene214, gene2274, gene2337, gene2609, gene2610, gene263, gene2711, gene2773, gene2954, gene3124, gene3502, gene3503, gene3547, gene3939, gene4046, gene4047, gene4048, gene4049, gene4081, gene4253, gene4488 |
| GO:0005975 | carbohydrate metabolic process | 120 min Down | 4.462E-06 | 0.0009735 | gene1001, gene1002, gene1120, gene116, gene1180, gene1378, gene1420, gene1449, gene1579, gene1612, gene1681, gene1698, gene1755, gene1896, gene1897, gene1898, gene1956, gene1958,gene1959, gene1960, gene1961, gene1968, gene1975, gene1978, gene2078, gene211, gene2119, gene2222, gene2223, gene2286, gene234, gene2472, gene2484, gene2519, gene2520, gene2524, gene255, gene257, gene258, gene2603, gene2679, gene2680, gene2681, gene275, gene2878, gene2902, gene2941, gene304,gen e3083, gene3127, gene3230, gene3233, gene3260, gene3370, gene353, gene3592, gene3595, gene3661, gene3662, gene3679, gene3869, gene4019, gene4056, gene42, gene4269, gene430, gene498, gene502, gene607, gene702, gene867, gene902, gene972 |
| GO:0005975 | carbohydrate metabolic process | 120 min Up | 4.462E-06 | 0.0009735 | gene1105, gene1761, gene1942, gene2351, gene2361, gene2365, gene2366, gene2368, gene2372, gene2376, gene2378, gene2379, gene2385, gene2386, gene2390, gene2404, gene2598, gene2795, gene3309, gene3311, gene3312, gene3331, gene3384, gene3412, gene3419, gene3420, gene3421, gene3481, gene3639, gene3641, gene3720, gene3934, gene4050, gene4220, gene4422, gene642, gene658, gene762 |
| GO:0006412 | translation | 120 min Down | 6.624E-06 | 0.0012387 | gene1071, gene1237, gene1526, gene177, gene2691, gene2951, gene35, gene3664, gene3956, gene4075, gene4101, gene4263, gene593, gene782 |
| GO:0006412 | translation | 120 min Up | 6.624E-06 | 0.0012387 | gene1159, gene1234, gene1235, gene2280, gene2310, gene2343, gene2344, gene2416, gene2417, gene2418, gene2419, gene2420, gene2421, gene2422, gene2423, gene2424, gene2425, gene2426, gene2428, gene2429, gene2430, gene2431, gene2432, gene2433, gene2434, gene2435, gene2436, gene2437, gene2439, gene2440, gene2441, gene2442, gene2487, gene2488, gene2578, gene2579, gene2666, gene2910, gene2922, gene3159, gene3160, gene3252, gene3263, gene3277, gene3352, gene3355, gene3608, gene3692, gene4032, gene4107, gene4216, gene4217, gene4219, gene4252, gene4257, gene4275, gene4338, gene4339, gene4340, gene4364, gene4472, gene4473, gene4474, gene4475, gene611 |
| GO:0043043 | peptide biosynthetic process | 120 min Down | 1.145E-05 | 0.0019992 | gene1071, gene1237, gene1526, gene177, gene2691, gene2951, gene3137, gene35, gene3664, gene3956, gene4075, gene4101, gene4263, gene593, gene782 |
| GO:0043043 | peptide biosynthetic process | 120 min Up | 1.145E-05 | 0.0019992 | gene1159, gene1234, gene1235, gene2015, gene2280, gene2310, gene2343, gene2344, gene2416, gene2417, gene2418, gene2419, gene2420, gene2421, gene2422, gene2423, gene2424, gene2425, gene2426, gene2428, gene2429, gene2430, gene2431, gene2432, gene2433, gene2434, gene2435, gene2436, gene2437, gene2439, gene2440, gene2441, gene2442, gene2487, gene2488, gene2578, gene2579, gene2666, gene2910, gene2922, gene3159, gene3160, gene3252, gene3263, gene3277, gene3352, gene3355, gene3608, gene3692, gene4032, gene4107, gene4216, gene4217, gene4219, gene4252, gene4257, gene4275, gene4338, gene4339, gene4340, gene4364, gene4472, gene4473, gene4474, gene4475, gene611 |
| GO:0006518 | peptide metabolic process | 120 min Down | 1.312E-05 | 0.0021473 | gene1071, gene1237, gene1526, gene177, gene2228, gene2691, gene2951, gene3137, gene35, gene3664, gene3956, gene4075, gene4101, gene4263, gene4398, gene593, gene782 |
| GO:0006518 | peptide metabolic process | 120 min Up | 1.312E-05 | 0.0021473 | gene1159, gene1234, gene1235, gene2015, gene2280, gene2310, gene2343, gene2344, gene2416, gene2417, gene2418, gene2419, gene2420, gene2421, gene2422, gene2423, gene2424, gene2425, gene2426, gene2428, gene2429, gene2430, gene2431, gene2432, gene2433, gene2434, gene2435, gene2436, gene2437, gene2439, gene2440, gene2441, gene2442, gene2487, gene2488, gene2558, gene2578, gene2579, gene2666, gene2910, gene2922, gene3159, gene3160, gene3252, gene3263, gene3277, gene3352, gene3355, gene3608, gene3692, gene4032, gene4107, gene4216, gene4217, gene4219, gene4252, gene4257, gene4275, gene4338, gene4339, gene4340, gene4364, gene4472, gene4473, gene4474, gene4475, gene611 |
| GO:0009081 | branched-chain amino acid metabolic process | 120 min Down | 5.256E-05 | 0.0077099 | gene1698, gene1755, gene2198, gene2222, gene2501, gene2502, gene2503, gene2504, gene2511, gene2512, gene2815, gene4055, gene4056, gene4057, gene4059, gene445, gene902 |
| GO:0043604 | amide biosynthetic process | 120 min Up | 7.539E-05 | 0.0098684 | gene1071, gene1078, gene1237, gene1526, gene177, gene1845, gene219, gene2306, gene2691, gene2951, gene3137, gene35, gene3511, gene3658, gene3664, gene3956, gene4075, gene4101, gene4263, gene593, gene782 |
| GO:0043604 | amide biosynthetic process | 120 min Up | 7.539E-05 | 0.0098684 | gene1159, gene1234, gene1235, gene2015, gene2280, gene2310, gene2343, gene2344, gene2416, gene2417, gene2418, gene2419, gene2420, gene2421, gene2422, gene2423, gene2424, gene2425, gene2426, gene2428, gene2429, gene2430, gene2431, gene2432, gene2433, gene2434, gene2435, gene2436, gene2437, gene2439, gene2440, gene2441, gene2442, gene2487, gene2488, gene2552, gene2578, gene2579, gene2596, gene2666, gene2910, gene2922, gene3088, gene3159, gene3160, gene3252, gene3263, gene3277, gene3328, gene3352, gene3355, gene3411, gene3503, gene3608, gene3692, gene4032, gene4107, gene4216, gene4217, gene4219, gene4252, gene4257, gene4275, gene4338, gene4339, gene4340, gene4364, gene4472, gene4473, gene4474, gene4475, gene611 |
| GO:0043603 | cellular amide metabolic process | 120 min Down | 8.555E-05 | 0.010665 | gene1071, gene1078, gene1220, gene1237, gene1526, gene177, gene1845, gene213, gene219, gene2228, gene2306, gene2691, gene2951, gene3137, gene35, gene3511, gene3599, gene3658, gene3664, gene3956, gene4075, gene4101, gene4244, gene4263, gene4398, gene593, gene633, gene635, gene782 |
| GO:0043603 | cellular amide metabolic process | 120 min Up | 8.555E-05 | 0.010665 | gene1159, gene1234, gene1235, gene2015, gene2280, gene2310, gene2343, gene2344, gene2416, gene2417, gene2418, gene2419, gene2420, gene2421, gene2422, gene2423, gene2424, gene2425, gene2426, gene2428, gene2429, gene2430, gene2431, gene2432, gene2433, gene2434, gene2435, gene2436, gene2437, gene2439, gene2440, gene2441, gene2442, gene2487, gene2488, gene2552, gene2558, gene2578, gene2579, gene2596, gene2666, gene2799, gene2910, gene2922, gene3088, gene3159, gene3160, gene3252, gene3263, gene3277, gene3328, gene3352, gene3355, gene3411, gene3503, gene3608, gene3692, gene3719, gene4032, gene4107, gene4216, gene4217, gene4219, gene4252, gene4257, gene4275, gene4338, gene4339, gene4340, gene4364, gene4472, gene4473, gene4474, gene4475, gene611 |
| GO:0009082 | branched-chain amino acid biosynthetic process | 120 min Down | 9.334E-05 | 0.0109667 | gene2198, gene2222, gene2501, gene2502, gene2503, gene2504, gene2511, gene2512, gene2815, gene4055, gene4056, gene4057, gene4059, gene445 |
| GO:0006099 | tricarboxylic acid cycle | 120 min Down | 0.0001047 | 0.0109667 | gene2484, gene3320, gene3675, gene3676, gene3677, gene3678, gene3679, gene3680, gene3681, gene3869, gene3870, gene4102, gene4103, gene4123, gene4132, gene869, gene990 |
| GO:0006099 | tricarboxylic acid cycle | 120 min Up | 0.0001047 | 0.0109667 | gene4241 |
| GO:0006101 | citrate metabolic process | 120 min Down | 0.0001047 | 0.0109667 | gene2484, gene3320, gene3675, gene3676, gene3677, gene3678, gene3679, gene3680, gene3681, gene3869, gene3870, gene4102, gene4103, gene4123, gene4132, gene869, gene990 |
| GO:0006101 | citrate metabolic process | 120 min Up | 0.0001047 | 0.0109667 | gene4241 |
| GO:0072350 | tricarboxylic acid metabolic process | 120 min Down | 0.0001047 | 0.0109667 | gene2484, gene3320, gene3675, gene3676, gene3677, gene3678, gene3679, gene3680, gene3681, gene3869, gene3870, gene4102, gene4103, gene4123, gene4132, gene869, gene990 |
| GO:0072350 | tricarboxylic acid metabolic process | 120 min Up | 0.0001047 | 0.0109667 | gene4241 |
| GO:0044262 | cellular carbohydrate metabolic process | 120 min Down | 0.0002387 | 0.0215447 | gene1001, gene1002, gene1420, gene1449, gene1956, gene1958, gene1959, gene1968, gene1975, gene1978, gene2222, gene234, gene2484, gene2519, gene2520, gene2524, gene255, gene257, gene2902, gene3127, gene3230, gene3233, gene3595, gene3869, gene4019, gene4056, gene42, gene502 |
| GO:0044262 | cellular carbohydrate metabolic process | 120 min Up | 0.0002387 | 0.0215447 | gene1942, gene2351, gene2361, gene2365, gene2366, gene2368, gene2372, gene2376, gene2378, gene2385, gene2390, gene2404, gene3331, gene3481, gene3639, gene3641, gene3720, gene4050, gene642, gene658 |
