# Supplementary material for: Temporal Transcriptional Responses of a Vibrio alginolyticus Strain to Podoviridae Phage HH109 Revealed by RNA-Seq
Source: mSystems. 2022 Apr 11;7(2):e00106-22. doi: 10.1128/msystems.00106-22 (PMC9040624; doi:10.1128/msystems.00106-22)
Supplement: TABLE S4 [file msystems.00106-22-s0004.docx]

**Table S4**

**Detailed KEGG pathways expression data on specific genes of *V. alginolyticus***

| **Pathway ID** | **Pathway** | ***P*-value** | **FDR** | **Group** | **Gene_list** |
| --- | --- | --- | --- | --- | --- |
| ko00230 | Nucleotide metabolism | 5.94698E-07 | 2.73561E-05 | 20 min Down | gene3503, gene1207, gene1240, gene2773, gene2226, gene3124, gene4081, gene4488, gene3502, gene3547, gene4080 |
| ko00230 | Nucleotide metabolism | 5.94698E-07 | 2.73561E-05 | 20 min UP | gene3039, gene3038 |
| ko00240 | Nucleotide metabolism | 0.004783609 | 0.110023007 | 20 min Down | gene2337, gene1728, gene3940, gene3939 |
| ko00240 | Nucleotide metabolism | 0.004783609 | 0.110023007 | 20 min UP | gene3039, gene3038 |
| ko02060 | Membrane transport | 0.010754042 | 0.146273254 | 20 min Down | gene256, gene3594, gene96, gene97 |
| ko00910 | Energy metabolism | 0.012719413 | 0.146273254 | 20 min UP | gene335, gene781, gene780, gene334 |
| ko00053 | Carbohydrate metabolism | 0.016630956 | 0.153004794 | 20 min Down | gene96, gene97 |
| ko00680 | Energy metabolism | 0.022807106 | 0.174854481 | 20 min UP | gene4137 |
| ko00680 | Energy metabolism | 0.022807106 | 0.174854481 | 20 min Down | gene2404, gene211, gene3413 |
| ko00624 | Xenobiotics biodegradation and metabolism | 0.042741935 | 0.228341993 | 20 min Up | gene876 |
| ko03420 | Replication and repair | 0.042815622 | 0.228341993 | 20 min Up | gene2266, gene3628 |
| ko03030 | Replication and repair | 0.044675607 | 0.228341993 | 20 min Up | gene4136, gene2266, gene3628 |
| ko03410 | Replication and repair | 0.090356123 | 0.415638166 | 20 min Up | gene2266, gene3628 |
| ko05132 | Infectious diseases: Bacterial | 0.001013251 | 0.122603334 | 60 min Up | gene397, gene4369 |
| ko05132 | Infectious diseases: Bacterial | 0.001013251 | 0.122603334 | 60 min Down | gene1757, gene4368, gene2698, gene3044, gene4298, gene1759, gene3101, gene1758 |
| ko00660 | Carbohydrate metabolism | 0.002244127 | 0.135769693 | 60 min Down | gene4059, gene2502, gene3682, gene2503, gene2511, gene2501, gene3681, gene2512, gene446 |
| ko00290 | Amino acid metabolism | 0.005366446 | 0.216446642 | 60 min Down | gene4059, gene4057, gene4055, gene2502, gene2503, gene4056, gene2511, gene2501, gene2512, gene2504, gene2198 |
| ko03010 | Translation | 0.017424166 | 0.527081013 | 60 min Up | gene1159, gene2417, gene2439, gene4217, gene2343, gene2421, gene4339, gene4216, gene3277, gene4474, gene2422, gene2420, gene2344, gene3160, gene2414, gene4472, gene2418, gene2416, gene4032, gene4473, gene2444, gene2547, gene2423, gene2424, gene4475, gene2425, gene2440, gene4219, gene2666, gene2922, gene2419 |
| ko04621 | Immune system | 0.022861823 | 0.553256124 | 60 min Up | gene397, gene4387 |
| ko04621 | Immune system | 0.022861823 | 0.553256124 | 60 min Down | gene3653, gene2698, gene4298, gene3101 |
| ko00260 | Amino acid metabolism | 0.028371385 | 0.553668706 | 60 min Up | gene3413, gene3013, gene631, gene632, gene3014, gene3031, gene2766, gene3083 |
| ko00260 | Amino acid metabolism | 0.028371385 | 0.553668706 | 60 min Down | gene3999, gene220, gene4001, gene4233, gene4055, gene2632, gene2631, gene2633, gene635, gene2173, gene1346, gene1844, gene2815, gene1982, gene1845, gene219 |
| ko02040 | Cell motility | 0.034143353 | 0.553668706 | 60 min Up | gene2711, gene2709, gene130, gene124, gene2708, gene2722, gene2710 |
|  |  |  |  | 60 min Down | gene4289, gene2700, gene4290, gene134, gene3573, gene3574, gene4287, gene4294, gene4288, gene2715, gene4293, gene1888, gene2698, gene2719, gene4298, gene2868, gene1866, gene133, gene3101, gene4291, gene4296, gene2702 |
| ko00362 | Xenobiotics biodegradation and metabolism | 0.036606195 | 0.553668706 | 60 min Up | gene2549, gene2363 |
| ko00362 | Xenobiotics biodegradation and metabolism | 0.036606195 | 0.553668706 | 60 min Down | gene907, gene876, gene1683, gene329, gene4007, gene4008 |
| ko00230 | Nucleotide metabolism | 0.05457496 | 0.733730015 | 60 min Up | gene3459, gene3039, gene2317, gene2773, gene3124, gene2319, gene4488, gene3502, gene263, gene4253, gene3503, gene2453, gene1093, gene3442, gene3547, gene3038, gene3671, gene1207, gene2274, gene1089, gene2451, gene4389, gene743, gene2454, gene4081 |
| ko00230 | Nucleotide metabolism | 0.05457496 | 0.733730015 | 60 min Down | gene1612, gene2570, gene3580, gene2941, gene4262, gene2568 |
| ko00261 | Biosynthesis of other secondary metabolites | 0.066306166 | 0.80230461 | 60 min Up | gene2453, gene2766, gene2454 |
| ko00261 | Biosynthesis of other secondary metabolites | 0.066306166 | 0.80230461 | 60 min Down | gene4001, gene2631, gene3112, gene2815 |
| ko03018 | Folding, sorting and degradation | 0.073816122 | 0.81197734 | 60 min Up | gene235, gene3460, gene4386, gene2917, gene4388, gene4330, gene4394, gene3384 |
| ko03018 | Folding, sorting and degradation | 0.073816122 | 0.81197734 | 60 min Down | gene144, gene3537, gene3465 |
| ko00333 | Biosynthesis of other secondary metabolites | 0.098775781 | 0.87416615 | 60 min Up | gene3712, gene2925, gene2926 |
| ko00333 | Biosynthesis of other secondary metabolites | 0.098775781 | 0.87416615 | 60 min Down | gene901 |
| ko03010 | Translation | 4.1667E-11 | 5.1667E-09 | 120 min Up | gene1159, gene2487, gene2437, gene2579, gene2417, gene4032, gene3352, gene4473, gene2439, gene4217, gene2343, gene2421, gene2488, gene4339, gene4216, gene2423, gene3277, gene2424, gene4474, gene2435, gene2434, gene2578, gene2422, gene2429, gene4475, gene2425, gene2428, gene2420, gene2344, gene4340, gene2433, gene2430, gene2440, gene3160, gene2441, gene4219, gene2426, gene2666, gene2432, gene3355, gene2922, gene2431, gene2442, gene4472, gene2419, gene2418, gene2436, gene2416 |
| ko00020 | Carbohydrate metabolism | 4.17066E-06 | 0.000258581 | 120 min Up | gene3341 |
| ko00020 | Carbohydrate metabolism | 4.17066E-06 | 0.000258581 | 120 min Down | gene4104, gene4102, gene990, gene4233, gene3679, gene3676, gene3320, gene4103, gene869, gene3677, gene2286, gene868, gene3675, gene3682, gene867, gene3680, gene3681, gene3089, gene4105, gene3678, gene2484, gene4132 |
| ko00720 | Energy metabolism | 0.000206283 | 0.007168122 | 120 min Up | gene4241, gene2896, gene2897, gene4139, gene4140 |
| ko00720 | Energy metabolism |  |  | 120 min Down | gene4104, gene907, gene4102, gene990, gene3676, gene4103, gene3320, gene3677, gene4137, gene3675, gene3682, gene3681, gene3089, gene211, gene4105, gene3678, gene4244, gene2484, gene4132 |
| ko00290 | Amino acid metabolism | 0.00023123 | 0.007168122 | 120 min Down | gene4059, gene4057, gene4055, gene2502, gene2503, gene445, gene4056, gene2511, gene2501, gene3755, gene2512, gene2504, gene2198 |
| ko00660 | Carbohydrate metabolism | 0.000318327 | 0.007894504 | 120 min Down | gene4059, gene2502, gene3682, gene2503, gene445, gene2511, gene2501, gene3681, gene2512, gene446 |
| ko00620 | Carbohydrate metabolism | 0.00663436 | 0.137110108 | 120 min Up | gene4241, gene2896, gene2897, gene3341, gene4139, gene4140 |
| ko00620 | Carbohydrate metabolism |  |  | 120 min Down | gene4104, gene2878, gene907, gene4, gene4102, gene797, gene4233, gene1834, gene4103, gene869, gene2941, gene1896, gene4137, gene2286, gene868, gene867, gene1556, gene3089, gene3137, gene211, gene4105, gene2504, gene2484, gene4132, gene3366 |
| ko00460 | Metabolism of other amino acids | 0.00802287 | 0.142119404 | 120 min Up | gene2558, gene2799 |
| ko00460 | Metabolism of other amino acids |  |  | 120 min Down | gene633, gene3599, gene213, gene4398 |
| ko00051 | Carbohydrate metabolism | 0.014128767 | 0.196924811 | 120 min Up | gene3419, gene2404, gene641, gene642, gene762 |
| ko00051 | Carbohydrate metabolism |  |  | 120 min Down | gene2525, gene2472, gene2524, gene257 |
| ko00640 | Carbohydrate metabolism | 0.014842488 | 0.196924811 | 120 min Up | gene2896, gene2897, gene4139, gene4140 |
| ko00640 | Carbohydrate metabolism | 0.014842488 | 0.196924811 | 120 min Down | gene2878, gene907, gene3868, gene4233, gene3870, gene653, gene3320, gene3869, gene2752, gene1896, gene4137, gene3872, gene3682, gene4007, gene2518, gene4009, gene3681, gene906 |
| ko00240 | Nucleotide metabolism | 0.016769683 | 0.196924811 | 120 min Up | gene3039, gene2136, gene3940, gene3038, gene2337, gene1531, gene2609, gene2661, gene743, gene81, gene1108, gene2610, gene1728, gene2954, gene3939, gene3158 |
| ko00240 | Nucleotide metabolism | 0.016769683 | 0.196924811 | 120 min Down | gene945, gene1810, gene3580, gene1241, gene3123, gene4262, gene2451, gene3259 |
| ko00430 | Metabolism of other amino acids | 0.017469136 | 0.196924811 | 120 min Up | gene2896, gene2897, gene1181, gene2558 |
| ko00430 | Metabolism of other amino acids | 0.017469136 | 0.196924811 | 120 min Down | gene1066, gene4398, gene1533 |
| ko00561 | Lipid metabolism | 0.019371417 | 0.200171307 | 120 min Up | gene3942, gene2923, gene4157, gene271 |
| ko00561 | Lipid metabolism | 0.019371417 | 0.200171307 | 120 min Down | gene2519, gene3233, gene1120, gene2173, gene2520, gene2518 |
| ko00030 | Carbohydrate metabolism | 0.022737793 | 0.216883568 | 120 min Up | gene4253, gene3419, gene2404, gene4220, gene3412, gene352 |
| ko00030 | Carbohydrate metabolism | 0.022737793 | 0.216883568 | 120 min Down | gene1612, gene353, gene2472, gene3258, gene2223, gene502, gene3260, gene2225, gene2222 |
| ko00500 | Carbohydrate metabolism | 0.031006388 | 0.264564177 | 120 min Up | gene1761 |
| ko00500 | Carbohydrate metabolism | 0.031006388 | 0.264564177 | 120 min Down | gene1612, gene3595, gene431, gene42, gene1001, gene1958, gene256, gene3594, gene4019, gene1959, gene1002, gene2902, gene1681, gene1960, gene257, gene3921, gene430, gene258 |
