# Supplementary material for: Temporal Transcriptional Responses of a Vibrio alginolyticus Strain to Podoviridae Phage HH109 Revealed by RNA-Seq
Source: mSystems. 2022 Apr 11;7(2):e00106-22. doi: 10.1128/msystems.00106-22 (PMC9040624; doi:10.1128/msystems.00106-22)
Supplement: TABLE S1 [file msystems.00106-22-s0001.docx]

Table S1

Details of the primer sequence used for qPCR.

| Primer pair | Sequence (5'-3') |
| --- | --- |
| HsdR F | AGTGTAAAGAGCAGTCACAGCATTG |
| HsdR R | GATCATCAACTGGTTGGTAAAGAAC |
| LysR F | ACTGCCATCTATGAAGAGCGTAACT |
| LysR R | GACACCTTTAACGCAACGATGAAAC |
| MFS F | TGAGGAAGCAATTCGAACTCAGATC |
| MFS R | AGTCCTTGTGCTTCCAATTGAACTA |
| OmpW F | ATCATGTAGTCCACACCAATATTCG |
| OmpW R | ATACGTTGGTGCAGGTCTGAACTAC |
| EF-Tu | TTGACGATGAAGAGCTACTAGAACT |
| EF-Tu | TTCCCACTGCTCTTCGCCGTTTAGT |
| RelE F | AAGAAGAGTCACTTAATGATCGTGA |
| RelE R | TGCCATCTCGTTGCACACCCATTAA |
| Asp F | TGTGGACTACATTGAGCAAGACAGA |
| Asp R | TAGTTGTTATCGAGTGGCAAGTTTC |
| HtpX F | ATTATGTTATTCCTAGCAACTAACC |
| HtpX R | ACCAAAACCAAATACAGCAGCCATT |
| FtsH F | GTGATTCCTGAAGGTGAGCGCGGTA |
| FtsH R | GTTGTTTCACACGGTCAAACAAAGG |
| TldD F | GAAGCGGTAGGTCATGGCCTAGAAG |
| TldD R | TGAACCACGAAGGTCTTTTAACGTG |
| AspA F | GTCGGTGAAACAATTTTGGATGTGC |
| AspA R | TTGAACGCGTACCGTTTTACCATTG |
| YccF F | CGTTTACAGCGCGACCGGGTATGTG |
| YccF R | GTAGTGTTGGTTTGCGCTGTGCTTG |
| NlpE F | CAGTAGAAGAAGAACAGGTAATTAC |
| NlpE R | ATACCAGCACAATCCGCGCAAGGTA |
| AmiB F | AGCGGAATTATTTAAATTAAGCCCA |
| AmiB R | AGCGGAATTATTTAAATTAAGCCCA |
| SapC F | GGCTCTCAGCTGACATTTGGCGCTG |
| SapC R | CATGACGGTATCGAGCAAGTGGTTG |
| OppB F | AATACCTTGATTACTCAGTAAACGA |
| OppB R | CTTGAGCGCAGCAATGGTACCGACG |
| MFS F | TGCGATCAACTTCCTGTGGGCACCT |
| MFS R | GGGTAGCGACGAGTACGATACTTTG |
| Mtp F | TCATGGTATCCAGTAGGATAGAGCC |
| Mtp R | CGAAATCCAATACCCAGTATCAGAA |
| YscG F | GATTCCTAATGTCCTGAGAAAAGCT |
| YscG R | CCTATCCAGATCTGATCAGCTTAGC |
| CesT F | CTTGAATAAATGCGTGTACGTCTGT |
| CesT R | GAAGTGAACTCACCAACATTGCTAA |
| FlaA F | AACGTTAATACTAACGTTTCTGCGA |
| FlaA R | TCTTTCGCGCTATTGATTTTATAAC |
| Tdh F | GTGCCTATGGTTGTCGGTCACGAAT |
| Tdh R | ACGACAATGACCACACGTGATGTGA |
| OppD F | ATATCTTCAGTGATGGAAGGAATCG |
| OppD R | GTATTGGTCATGTATGGTGGTCAAA |
| SapB F | GGGTGGCTTTGTTACTTATCATGGT |
| SapB R | CAAATCCGGTTACATGATCAATTTC |
| PBP F | ACCTCTTTCTTCGCATTGTTTTCAG |
| PBP R | AGTACTAGAAAATGCCGGTGGTGGT |
| gp35 F | GAAGAGCGTATCAATGCTATTCGAG |
| gp35 R | CGTACTGCATTGACCAGTAACGAGC |
| gp01 F | AAGGACACAATCGCAAACCTTCTAA |
| gp01 R | CACGCATGAACGACTCTTTGCTCAT |
| gp37 F | AGATGGTCTGATAAGTTGGATGAGG |
| gp37 R | ACCAGTACTGCGTAGACGAACAACT |
| DnaK_F | TAAACCCTGACGAAGC |
| DanK_R | AGTCATCACGCCACCC |
